# Hepatitis C new drugs

**DOI:** 10.1186/1758-2652-13-S4-O29

**Published:** 2010-11-08

**Authors:** H Wedemeyer

**Affiliations:** 1 Hannover Medical School, Dept. of Gastroenterology, Hepatology and Endocrinology, Hannover, Germany

## 

Hepatitis C virus infection remains a significant global health problem with more than 130 Million individuals being chronically infected world-wide. Since 2001, the standard therapy of chronic hepatitis has been PEG-IFNa + ribavirin. A large number of new direct acting antiviral agents is currently explored in clinical trials. Different viral proteins are targeted by these novel agents. The first HCV protease inhibitors telaprevir and boceprevir are expected to be approved in 2011 as phase III trials have been completed in summer 2010. At this stage, Direct Acting Anti-Viral agents will only be used in combination with PEG-IFNa and ribavirin. Triple therapy will increase sustained virological response rates by 25-30% for treatment naïve patients infected with HCV genotype 1 to 70-80% and previous nonresponder patients may now have chance of 30-50% to cure the infection. Moreover, treatment will be “response guided” meaning that patients who are already HCV RNA negative by week 4 can be treated shorter for only 24-28 weeks while slow responder still have to treated for 48 weeks. Resistance will be a problem for first generation HCV protease inhibitors, in particular if only suboptimal doses of PEG-IFNa and ribavirin can be administered. Finally, the new drugs will add additional side effects as telaprevir may cause rashes in 5-10% of patients and boceprevir can induce nausea. Both drugs can induce anaemia which may be more pronounced for boceprevir.

Additional drugs including “second wave protease inhibitors”, nucleosidic and non-nucleosidic polymerase inhibitors, NS5A inhibitors as well as cyclophilin inhibitors are currently explored in phase II studies. All oral therapies without PEG-IFNa are also explored by different companies. In addition therapeutic vaccine trials aiming to induce immune control are also still ongoing. It is very likely that the entire treatment concept for HCV infection will completely change within the next 5 years. Additional challenges will not only include management of viral resistance but also management of new side effects which may become of particular importance in patients with advanced liver disease.

